# Preliminary Analysis of Several Attractants and Spatial Repellents for the Mosquito, Aedes *albopictus* using an Olfactometer

**DOI:** 10.1673/031.012.7601

**Published:** 2012-07-05

**Authors:** Huiling Hao, Jingcheng Sun, Jianqing Dai

**Affiliations:** ^1^ Naval Medical Research Institute, 880 Xiangyin Road, Shanghai, 200433, China; ^2^ Guangdong Entomological institute, 105 Xingang Road, Guangzhou, 510260, China

**Keywords:** behavioral responses, testing conditions

## Abstract

Mosquito attractants and spatial repellents hold great promise in controlling mosquito pests. In assessing the effectiveness of mosquito attractants and repellents, a good olfactometer system, and optimized testing conditions, are essential. In this research, we demonstrated the usefulness of an olfactometer system, and optimized testing conditions for *Aedes albopictus* (Diptera: Culicidae). We found no significant difference in the biting activity of the insect between 8:00 and 22:00. Furthermore, 5–10 day old mosquitoes were relatively strongly attracted, with bloodsucking rate 75.8%. The random capture rate (entered trap in absence of odor) was less than 20% for the 20–40 tested insects. Capture rates and systematic errors increased as the number of testing insects increased. Wind speed affected the capture rate significantly, whereas variations in temperature between 25–28°C did not result in significant difference. The wind speed of 0.2 m/s exhibited a higher capture rate, which was significantly different from those at either 0.1 m/s or 0.4 m/s (*P* < 0.05). At the wind speed of 0.2 m/s, time durations within the first 8 minutes correlated positively with capture rates (r^2^ = 0.997), but further increase in time duration to 10 minutes did not result in further increase in capture rates. One percent of L-lactic acid in dichloromethane resulted in a consistently higher capture rate (43.8%) than that from a human odor (31.2%). Under our testing conditions, eugenol, anisaldehyde, geraniol, citronellal, citral, and linalool all exhibited some inhibition effect on mosquitoes to successfully trace human odor or 1% of L-lactic acid in dichloromethane. The results of these two tests indicate that the L-lactic acid/dichloromethane mixture may be used as an effective attractant to evaluate the effect of possible spatial repellents on *Ae. albopictus*.

## Introduction

The Asian tiger mosquito, *Aedes albopictus*, is a very important pest to humans and animals. Although it is native to tropical and subtropical regions in Southeast Asia, *Ae. albopictus* is successfully adapting itself to cooler regions (Hawley et al. 1989), and has spread to Europe, the Americas, the Caribbean, Africa, and the Middle East (Ritchie 2006; Pluskota et al. 2008). *Ae. albopictus* can transmit various pathogens, such as the West Nile virus, Yellow fever virus, St. Louis Encephalitis, Dengue fever, and Chikungunya fever (Hochedez et al. 2006). Like other mosquitoes, *Ae. albopictus* is very difficult to suppress or control due to their remarkable ability to adapt to various environments, their close contact with humans, and their reproductive biology. Application of chemical pesticides against adult mosquitoes has only a limited effect. Pesticide application has also been an increased concern for environmental contamination (Sukumar et al. 1991).

In the search for effective alternatives to pesticides in managing mosquitoes, a great deal of attention has been paid to mosquito attractants and repellents. Many synthetic and natural volatile organic compounds possess the ability to either attract or repel adult mosquitoes (Fradin 1998; Fradin and Day 2002). Arrays of attractant-based traps have been used as protective barriers in large areas, and as killing stations in backyards by home owners (Kline and Lemire 1998). To further increase the effectiveness of attractant-based traps, the application of combined attractants and repellents, namely attractant-based traps together with a repellent barrier, has been proposed in managing mosquitoes (Kline et al. 2003). Towards this goal, the effectiveness of various attractants and other volatile organic compounds that may serve as spatial repellents have been tested extensively against the mosquito species *Ae. aegypti* (Carroll 1979; Geier et al. 1996; Bernier et al. 2003; Kline et al. 2003). Recently, the effects of several plant-based organic chemicals including geraniol, citral, citronellal, eugenol, and anisaldehyde on host-seeking and bloodsucking behavior of *Ae. albopictus* have been evaluated (Hao et al. 2008). Very limited information, however, is available on the effectiveness of attractants and spatial repellents to *Ae. albopictus* (Trexler et al. 2003). Our goal is to establish a management method proposed by Kline et al (2003) using a combination of attractants and spatial repellents for the control of *Ae. albopictus*. Towards this goal, we need to screen and evaluate a large number of candidate chemicals under various experimental conditions. The objectives of this research are to characterize *Ae. albopictus* biting behavior, to establish an effective olfactometer system and test its applicability, to optimize testing conditions with various biological and environmental parameters, and to initially analyze the effectiveness of several attractants (Bernier et al. 2003) and spatial repellents (Hao et al. 2008) against *Ae. albopictus* under optimized conditions.

## Materials and Methods

### Insect

*Ae. albopictus* were originally obtained from a colony at the China Institute of Entomology. They were maintained in our laboratory at 25 ± 1° C , 70% RH, a photoperiod of 14:10 L: D, and fed a 6% glucose solution diet. This strain has been in continuous culture for more than ten years.

### Chemicals and reagents

L-Lactic acid (> 99%) was from Sigma (St. Louis, MO, USA). Dichloromethane (> 99.5%) and acetone (>99%) were from Sinopharm Chemical Reagent Co. (Shanghai, China). Eugenol (99%, CAS-No.97-53-0) and anisaldehyde (99%, CAS-No. 123-11-5) were from Sinopharm Chemical Reagent Co. (Shanghai, China). Geraniol (96%, CAS-No. 106-24-1), citronellal (96%, CAS-No. 10623-0), citral (97%, CAS-No.5392-40-5), and linalool (98%, CAS-No. 78-70-6) were donated by the Shanghai Apple Flavor & Fragrance Co., Ltd.

### Observation of *Ae. albopictus* biting behavior

*Ae. albopictus* nulliparous female adults were divided into two age groups in our experiment: 5–10 days old insects, and 10–20 days old insects. An arm-in-cage test was used to assay biting ability. Individual mosquitoes were isolated in a gauze cage (300 × 300 × 300 mm) and allowed to orient toward the hand and forearm during a 2 minute test period. The host-seeking and biting ability of *Ae. albopictus* was defined as the ability of mosquitoes to locate the target, land, and search for a suitable site before insertion of its stylets into the arm. Biting behaviors were conducted four times each day, at 8:00, 13:00, 18:00, and 22:00, respectively. Each observation lasted 20 minutes, and each experiment was repeated 30 times.

### The olfactometer system

**Figure 1.**
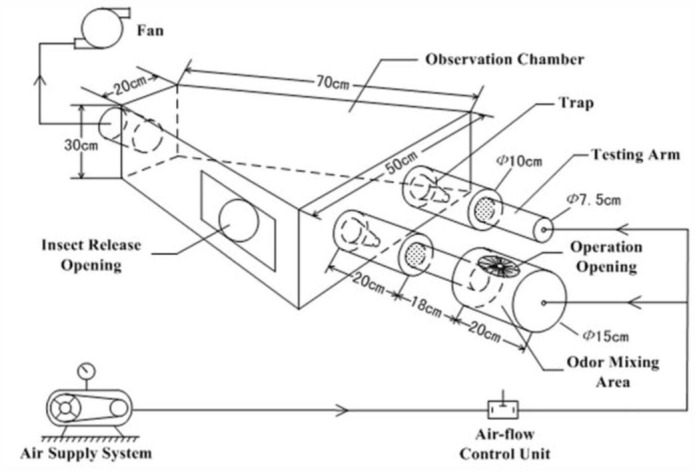
A schematic representation of the olfactometer. High quality figures are available online.

The olfactometer was self-made, and is shown in Figure 1. The device consists of an insectreleasing opening on the front right side. On the front side, there are two testing arms where chemicals for testing are placed, and a trap on the back of each arm. An exit opening is located on the back of the device. The system is equipped with a filtered external air supply system that has temperature and relative humidity control.

**Figure 2.**
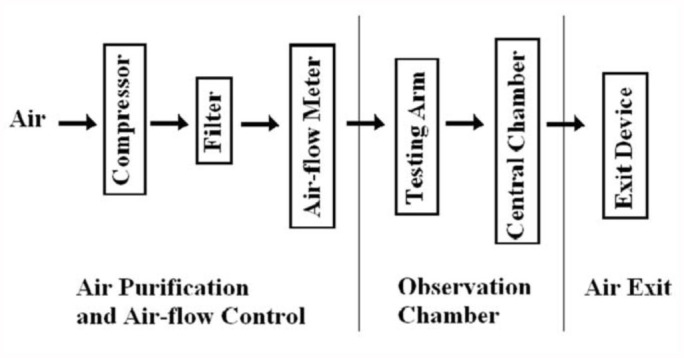
A schematic presentation of air-flow and testing. High quality figures are available online.

The assay was conducted as shown in Figure 2. Briefly, air was pumped by a compressor through an air-filter. The air-flow was measured by an Air-Flow Meter before it was released into the olfactometer chamber. When the air was passing through the testing arm of the olfactometer, where attractants or repellents were placed, the air mixed with attractants or repellents, and brought them into the center of the test chamber. The air was expelled out by an 18 Watt fan through an exit opening.

When *Ae. albopictus* female adults were exposed to attractants and repellents in the test chamber of the olfactometer, responses and behavior changes were examined. If the insects were sensitive to certain attractants or repellents, they would react differently. In the case of an attractant, most of the *Ae. albopictus* female adults initiated flight and migrated to the attractants. The difference in behavioral responses to different chemicals should be reflected in the numbers of insects caught by the trap. By comparing the number of insects to fall into the trap, and the amount of time it took for them to do so, we could infer the sensitivity of the insects to the attractants or repellents. Observations were conducted after the system was on for 30 minutes. Test mosquitoes were allowed to acclimatize for 15 minutes before initiating the bioassay.

### Optimizing testing conditions for the olfactometer

Different insect densities (20, 40, 60, and 80 insects per observation), temperatures (25, 26, 27, and 28° C), air-flow rates (0.1, 0.2, and 0.4 m/s), and time durations (2, 4, 6, 8, and 10 minutes) were evaluated for the effect on capture efficiency. Each experiment was repeated four times, and 5–10 days old nulliparous female adults were used.

### Optimizing conditions for chemical attractants with the olfactometer

When examining spatial repellents, non-live (chemical) attractants were often used because of their consistency in comparison with live attractants. During the screening with chemical attractants, 300 µl solution of candidate attractant in a 400 µl glass container was used. It was placed into one of the test arms, while the other test arm contained a non-attractant control. The number of insects that fell into the trap (captured) was recorded after 8 minutes of testing. As a live attractant, the operator's hand was exposed through the screen at one of the test arm ports for a duration of 8 minutes, while the other test arm served as a control. The device was cleaned after each experiment before the next use. Each experiment was repeated five times, and 5–10 days old nulliparous female adults were used

### Spatial repellency of compounds against *Ae. albopictus*

The experiment was carried out in two groups, chemical attractants and human odor. As described in the chemical attractant test, a glass dish containing 300 µl of 1% L-lactic acid in dichloromethane was placed in both arms of the olfactometer. At the same time, one arm also contained 10 µl of one of the testing chemicals (eugenol, anisaldehyde, geraniol, citronellal, citral, and linalool), while the other arm served as a control. Data were also collected with exchange of the test and control arms. In the human odor test, one hand was placed in each arm, while one arm contained 10 µl of one of the testing chemicals described above. Again, data were collected with exchanges of the arms to avoid bias. Each experiment was repeated three times, and 5–10 days old nulliparous female adults were used.

All the experiments were carried out at 70 ± 5% relative humidity, with 4500 lux light intensity, unless otherwise stated.

### Data analysis

One-way ANOVA and DUNCAN test were used in the analysis of data on *Ae. albopictus* biting behavior, impact of temperature, and the effectiveness of attractants. Linear regression was used in the analysis of the relationship between observation duration and the number of insects trapped.

## Results

### Effect of photoperiod on the biting behavior

**Figure 3.**
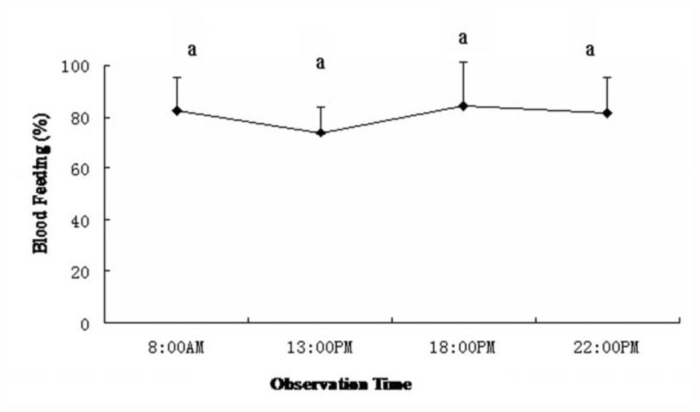
Blood-feeding activity of *Aedes albopictus* during the day. High quality figures are available online.

The effect of photoperiod on the biting behavior of the *Ae. albopictus* strain used in this study is shown in Figure 3. *Ae. albopictus* female adults did not exhibit significant changes in biting behavior between 8:00 and 22:00. The *Ae. albopictus* strain used in this study was naturally light-insensitive. *Ae. albopictus* is less sensitive to light than *Ae. aegypti* under natural conditions (Kawada et al. 2006). This observation suggested that light conditions have little effect on biting behavior, so tests of attractants and repellents with *Ae. albopictus* female adults could be conducted at any time of a day.

### Effect of insect age on biting behavior

**Figure 4.**
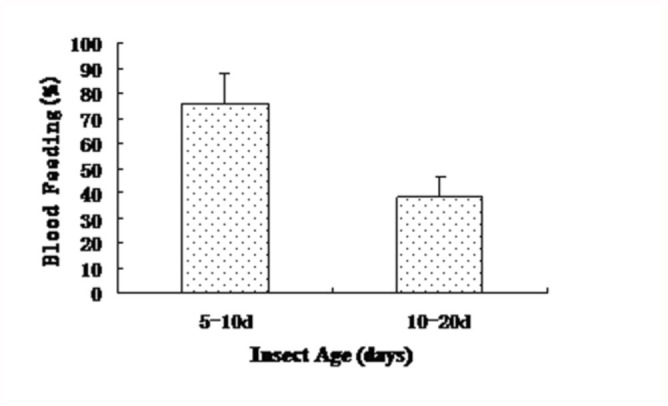
Blood-feeding behavior of *Aedes albopictus* at different ages. High quality figures are available online.

There was a significant difference in biting behavior between the two age groups of *Ae. albopictus* female adults (Figure 4). Within 20 minutes, 75.8% of 5–10 days old female adults exhibited biting and blood-sucking, while only 37.9% of 10–20 days old female adults exhibited biting and blood-sucking. The decline with age in the insect's ability to attack suggested that 5–10 days old female adults would be more suitable for experimental assays.

### Effect of insect density on random capture rate

In this study, random capture refers to the mosquitoes entering the trap in the absence of odor. As shown in Table 1, the percentage of random capture increased as insect density became higher. When 20 insects were released into the olfactometer chamber, the random capture was 6.0%. As the number of insects increased to 40, the random capture increased to 16.5%. With further increases in insect density to 60 and 80 insects, random capture increased to 25.6% and 40.3%, respectively. These results meant insect density of 20 may prevent 94% mosquitoes from entering the collection arms without stimulus, and the systemic error at insect density of 20 was 6.0%.

### Effect of temperature on capture rate

Since temperature might affect the capture efficiency, we examined random capture rate on *Ae. albopictus* female adults under four different temperatures, namely 25, 26, 27, and 28° C (Table 2). In each case, 20 insects of 5–10 days old were used. Our data indicated that within 25 to 28° C, temperature variation had no apparent effect on insect capture under our experimental conditions.

### Effect of air-flow and time-length on capture rate

**Figure 5.**
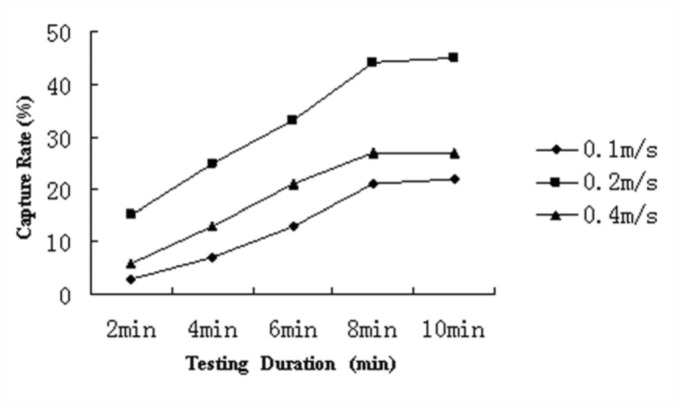
Relationship between capture rate and testing duration at three different air-flow speeds (0.1, 0.2, and 0.4 m/s). High quality figures are available online.

As shown in Figure 5, capture rates at air-flow 0.1 m/s, 0.2 m/s, and 0.4 m/s was significantly different (*p* < 0.05) from each other. The capture rate was higher with air-flow of 0.2 m/s. The capture rate was also highly correlated with time lengths under each airflow speed. At all the three air-flow speeds, the capture rates increased linearly between 2 and 8 minutes, with correlation coefficients (r^2^) 0.985, 0.997, and 0.995, respectively, between time duration and number of insects captured. However, no significant differences in capture rates were observed between 8 and 10 minute durations.

### Effect of different attractants on capture rate

After determining the optimum conditions of the insect (age and density) and environmental factors (temperature, air-flow speed, and observation time) for testing, different attractants were screened at different concentrations for the effectiveness of insect attraction under the following optimum conditions: 20 5–10 days old nulliparous females at 25–28° C, 0.2 m/s air-flow speed, and 8 minute observation duration. The testing combinations of different attractants at different concentrations are given in Table 3. In single chemical tests, L-lactic acid, acetone, and dichloromethane had the average capture rates of 10.1%, 15.3%, and 25.5%, respectively. Under the stated experimental conditions, dichloromethane alone was as high as human odor, which had an average capture rate of 31.2% (not statistically different from that of dichloromethane). On the other hand, L-lactic acid and acetone were less effective in their attraction of *Ae. albopictus* female adults, with capture rates 10–15% lower than the capture rate of human odor. The results of the experiment demonstrated that the combinations of L-lactic acid and dichloromethane, and of L-lactic acid and acetone, were much more effective in their attraction of *Ae. albopictus* female adults than the three chemicals were by themselves. A mixture of 1% L-lactic acid in acetone resulted in a capture rate of 40.2%, and is comparable with a mixture of 1% L-lactic acid in dichloromethane, which resulted in a capture rate of 43.8%. Further increases of L-lactic acid to 10% in both acetone and dichloromethane did not result in significant increases in capture rates.

**Table 1.**
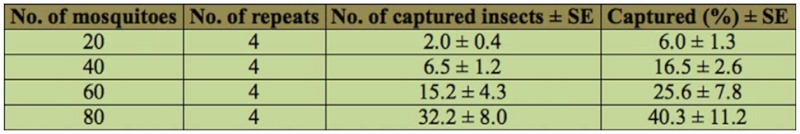
Capture rates with different insect densities under control conditions.

**Table 2.**
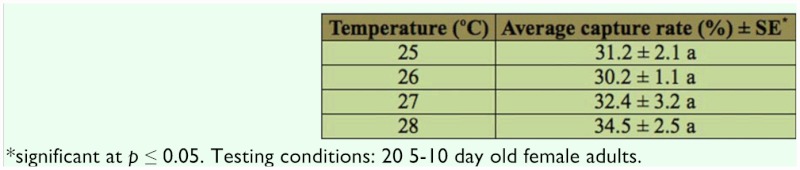
Effect of temperature on attraction of *Ae. albopictus* to a human skin.

**Table 3.**
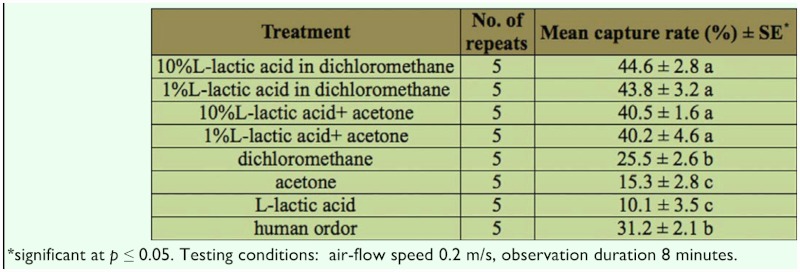
Capture rates of *Ae. albopictus* by various chemicals and chemical combinations.

### The effect of spatial repellents against *Ae. albopictus*

**Figure 6.**
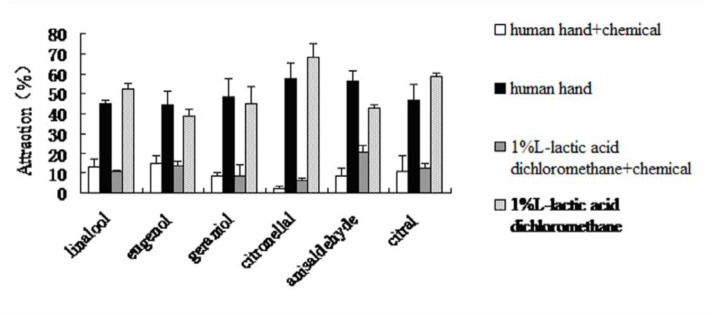
Inhibitory effect of repellents on the response of *Aedes alpopictus* to chemical attractants. High quality figures are available online.

Under optimum conditions (20 5–10 days old females, at 25–28° C, 0.2 m/s air-flow speed, and 8 minute duration), the six tested chemicals all exhibited inhibitory activity to the reception of chemical attractants and human odor by mosquito females (Figure 6). In the testing arm with the chemical, only a small number of female adults were captured.

Mosquito adults flew towards the other arm against air-flow, exhibiting various degrees of avoidance. In all six groups of testing, the results with chemical attractants were generally in agreement with the results with human odor, indicating that chemical attractants may be used as effective non-live attractants for future research.

## Discussion

Mosquito attractants and spatial repellents hold great promise in managing mosquito populations, which would reduce the spread of infectious diseases. Various volatile organic compounds possess the ability to inhibit the scent orientation of insects in an environmentally defined three dimensional space, and therefore can be potentially used as spatial repellent in managing mosquito populations (Dogan and Rossignol 1999; Fradin and Day 2002; Nolen et al. 2002; Oyedele et al. 2002; Kline et al. 2003; Achee et al. 2009; Drapeau et al. 2009). However, the effectiveness of any attractant or repellent must be carefully evaluated before practical application. Different mosquito species, or even different geographic subpopulations of a given species, may react differently to a given chemical. Currently, most tests of attractants and potential spatial repellents have been focused on the mosquito *Aedes aegypti* (Carroll 1979; Geier and Boeckh 1999; Bernier et al. 2003; Kline et al. 2003). The effectiveness of attractants and repellents has not been systematically evaluated under well-controlled conditions including insect densities, temperatures, air-flow rates, and time durations. The influence of mosquito age and density on repellent tests has been reported previously (Barnard et al. 1998); however, the method used was a contact repellency bioassay in a cage, but not spatial repellency bioassay in a wind tunnel bioassay system, and the test species were *Ae. aegypti* and *Anopheles quadrimaculatus*. In our study, *Ae. albopictus* female adults did not exhibit significant changes in its biting behavior between 08:00 and 22:00. But, the study of Xue and Barnard (1996) showed the distribution of mean host attack responses during the dial period was bimodal, with approximately 70% of all activity during the photophase (08:00–20:00). Attack rates were highest in the morning and evening. This might be due to the fact that the *Ae. albopictus* strain had been maintained in the laboratory for many generations, and its photoperiod cycle had decayed. Alternatively, the *Ae. albopictus* strain used could be naturally light-insensitive.

In this research, a newly constructed olfactometer system that can be useful for evaluation of mosquito attractants and repellents was demonstrated. Biological and environmental parameters were optimized for effective analysis of attractants and repellents against *Ae. albopictus* female adults. Several potential repellents were analyzed for their effectiveness under certain experimental conditions. The next step in this research is to systematically and comparatively analyze various potential combinations of attractants and spatial repellents. The availability of these results should provide useful information for developing attractant-based traps with a spatial repellent barrier (Kline et al. 2003).

In an initial analysis, the effectiveness of each of the three attractants, L-lactic acid, acetone, and dichloromethane, was compared against each other. All three chemicals were effective in their attraction of *Ae. albopictus* female adults. However, the effectiveness of these three chemicals varied greatly. Among them, dichloromethane was most effective, resulting in a capture rate comparable to that of human odor. On the other hand, L-lactic acid, a major metabolite of human skin, resulted in only a 10% capture rate, the lowest among all the three chemicals. The effectiveness of combinations of these three chemicals was dramatically different from that of the single chemicals. When 1% of lactic acid was added to either acetone or dichloromethane, the capture rate increased to over 40%, higher than human odor. Clearly, the gradients, and the proportion of the gradients, in a mosquito cue are important in its effectiveness. The repeatability of chemical testing in the experiment indicated that our olfactometer system, optimized testing conditions, and the selected chemical combinations would be effective in future mosquito attractants and repellents research.
